# Texture Analysis of Histology Images for Characterizing Ultrasound-Stimulated Microbubble Radiation Enhancement Treatment Response

**DOI:** 10.3390/cells14242023

**Published:** 2025-12-18

**Authors:** Lakshmanan Sannachi, Serena Mohabir, Evan McNabb, Deepa Sharma, Anoja Giles, Wenyi Yang, Kai Xuan Leong, Martin Stanisz, Gregory J. Czarnota

**Affiliations:** 1 Physical Sciences Platform, Sunnybrook Research Institute, Sunnybrook Health Sciences Centre, Toronto, ON M4N 3M5, Canada; lakshmanan.sannachi@sunnybrook.ca (L.S.); martin.stanisz@sunnybrook.ca (M.S.); 2 Department of Radiation Oncology, Odette Cancer Centre, Sunnybrook Health Sciences Centre, Toronto, ON M4N 3M5, Canada; 3 Department of Medical Biophysics, University of Toronto, Toronto, ON M4N 3M5, Canada; 4 Department of Radiation Oncology, University of Toronto, Toronto, ON M4N 3M5, Canada

**Keywords:** texture derivative, microbubbles, radiation enhancement, MRI-guided focused ultrasound, histology, radiomics

## Abstract

Ultrasound-stimulated microbubble (USMB) therapy, in combination with radiotherapy (XRT), represents a promising approach to enhancing the efficacy of conventional cancer treatments by targeting tumor vasculature. Recent preclinical studies using MRI-guided focused ultrasound have demonstrated that USMB enhances radiation effects in tumor blood vessels, resulting in significantly greater tumor cell death than radiation alone. Dynamic contrast-enhanced MRI (DCE-MRI) has been instrumental in this methodology in mapping tumor perfusion heterogeneity, allowing for precise targeting of additional USMB and XRT to specific vascular regions. This study employed four advanced texture analysis methods, GLCM, GLDM, GLSZM, and NGTDM, to quantitatively assess changes in the cellular structure of prostate tumors following different treatments, including combinations of USMB and XRT targeted to low- and high-perfusion regions. Texture features, particularly those derived from GLCM, GLDM, and GLSZM, revealed significant differences in cell structure patterns across treatment groups. The GLSZM methodology was identified as the most sensitive method for detecting treatment-induced structural changes, effectively identifying regions of necrosis and varied stages of cell death. Texture-derivative analyses further highlighted intra-tumoral heterogeneity, especially in response to additional USMB + XRT treatments. These results align with findings in other tissue models, underscoring the value of texture analysis for monitoring treatment response.

## 1. Introduction

Radiotherapy remains a cornerstone in the management of various malignancies, leveraging ionizing radiation to induce DNA damage and eradicate tumor cells. The precision of modern radiotherapy has been greatly enhanced by the integration of advanced imaging modalities. Techniques such as ultrasound, computed tomography, and magnetic resonance imaging (MRI) are now routinely employed to achieve accurate tumor targeting and minimize collateral damage to surrounding healthy tissues [1,2,3,4]. Compared to CT-based guidance, MRI and ultrasound are non-ionizing imaging modalities that enable repeated, high-frequency imaging without additional radiation exposure. This makes them ideal for longitudinal monitoring, adaptive treatment planning, and response assessment—particularly in sensitive or preclinical models. Their ability to provide frequent, longitudinal guidance in image-guided radiotherapy (IGRT) supports safer adaptive workflows and biologically informed dose modulation. In particular, MRI provides superior soft tissue contrast without ionizing radiation exposure, making it highly effective for accurate tumor delineation and for acquiring biological and functional information from both tumors and adjacent normal tissues [5,6]. MRI has thus become an essential tool for identifying clinical target volumes and organs at risk during radiation planning. Clinical studies have demonstrated that MR-guided radiation is feasible and effective and can significantly improve patient outcomes in the treatment of cancers such as nasopharyngeal carcinoma, lung tumors, breast cancer, pancreatic cancer, and prostate cancer [7].

Despite advances in radiation delivery, high-dose radiation typically administered as 70 Gy over 35 fractions in combination with chemotherapy remains the standard for effective tumor control while minimizing damage to adjacent healthy tissues. Chemotherapy acts as a radiosensitizer, augmenting the effects of radiation and improving therapeutic outcomes [8,9,10]. Nevertheless, locoregional relapse remains the most frequent pattern of failure, and high-dose radiation is associated with substantial short- and long-term adverse effects. These challenges underscore the need for strategies that enhance RT efficacy while reducing radiation doses.

One promising approach involves the use of high-intensity focused ultrasound (HIFU) as an adjunct to radiation treatment (RT). Focused ultrasound (FUS) offers high spatial precision, enabling selective targeting of tumor tissue while sparing surrounding normal structures [11,12,13]. Preclinical studies have demonstrated that combining FUS with RT can significantly enhance tumor cell death and improve overall therapeutic efficacy [11,12]. Recently, a novel combined treatment strategy has emerged, wherein intravenously administered microbubbles are stimulated with ultrasound to perturb tumor vascular endothelial cells, thereby enhancing radiation effects [14]. Upon ultrasound exposure, microbubbles oscillate and cavitate, transferring mechanical stress to adjacent endothelial cell membranes. This mechanical effect sensitizes endothelial cells to radiation through a membrane-activated, ceramide-mediated cell death pathway—a process that typically requires very high single doses of radiation. Preclinical studies have shown that ultrasound-stimulated microbubble (USMB) treatment, when combined with low-dose radiation, results in significant tumor size reductions. Further investigations have elucidated the extent of endothelial disruption in vitro and identified potential gene markers and signaling pathways implicated in this response. Multiple in vivo studies have demonstrated the effectiveness of this approach in treating xenograft tumors in murine models, including breast, prostate, liver, and bladder cancers [15]. Our previous work demonstrated the feasibility of ultrasound image-guided USMB treatment using a breast cancer xenograft model. Building on these findings, we recently demonstrated the radiation enhancement effect of USMB treatment in a larger and more clinically relevant xenograft prostate tumor model in rabbits. More recently, we have explored the use of a commercial MRI-guided focused ultrasound system (Sonalleve V2; Profound Medical, Toronto, Canada) in combination with human prostate cancer (PC3) xenografts in rabbits [16]. This study reported a 5% increase in tumor cell death when combining RT with USMB therapy.

In addition, dynamic contrast-enhanced MRI (DCE-MRI) was used to identify low- and high-perfusion areas within tumors, which were then selectively treated with additional combined USMB and RT. Histological analysis using Factor VIII staining revealed a greater decrease in vascular density following the combined treatment. The treatment groups investigated in this study included control, radiotherapy alone, USMB alone, combined USMB and radiotherapy, and additional combined treatment targeted to low- and high-perfusion regions. The effects of each treatment type on tumor cellular structure were assessed using histological imaging, which revealed varying degrees of tumor cell damage. To quantitatively assess changes in tumor cellular structure induced by different treatment modalities, we employed radiomics texture analysis of histology images.

Radiomics texture analysis has been widely applied to images obtained via multiple modalities, including MRI, CT, ultrasound, and histopathology [17,18,19,20]. In prostate cancer research, texture analysis of MRI and PET-CT images has been used for tumor characterization and to predict clinical treatment outcomes [21,22]. Notably, radiomics features such as the gray-level run-length matrix (GLRLM)-, gray-level zone length matrix (GLZLM)-, gray-level co-occurrence matrix (GLCM)-, and neighborhood gray-tone difference matrix (NGTDM)-based features extracted from MRI scans of prostate cancer patients undergoing radiotherapy have been shown to predict early structural changes in the femoral heads due to radiation exposure [23]. In another study, texture analysis of PET/CT images enabled accurate differentiation between metastatic lesions and completely responded sclerotic areas with an accuracy of 74% in prostate cancer patients after treatment [24]. Additionally, MRI-based radiomics models have been developed to predict prostate-specific antigen response in patients with metastatic castration-resistant prostate cancer following abiraterone acetate therapy, with an AUC of 0.88 [25].

Similar to other modalities, texture analysis of histology images has been proposed as a prognostic tool for treatment management in several cancers. For example, gray-level co-occurrence matrix (GLCM) analysis of histology images has been used to develop accurate prognostic tools for breast cancer, with studies reporting that combining clinical pathological and texture variables into a composite score improves prognostic performance, achieving accuracies of up to 90% [26]. More recently, texture analysis has been used to identify brain tumor grade and customize treatment, with reported accuracies of 95.8% using methods such as local binary patterns (LBPs), GLCM, combined LBGLCM, and gray-level run-length co-occurrence matrix (GLRLCM) analysis [27]. In a histology image-based tissue characterization study, PyrRadiomics texture analysis showed promising results in distinguishing non-malignant from malignant breast lesions, achieving classification rates of 100% and 80%, respectively [19]. In another study, a histology image-based radiomics model was developed to differentiate various tissue types, particularly separating tumors from stromata in colorectal cancer histology images, with an accuracy of 98.6% [20]. In our laboratory, texture analysis has also been performed on quantitative ultrasound parametric images for breast lesion characterization and for predicting breast cancer treatment response prior to therapy initiation [28]. In addition to standard radiomics texture analysis, we have derived texture-derivative parameters from parametric maps using a sliding window approach, demonstrating further improvements in tumor response prediction [29]. This is based on the hypothesis that second-order texture-derivative parameters better reflect intra-tumoral heterogeneity than fundamental textural parameters, thereby improving the prediction of clinical outcomes. 

In the present study, we investigated changes in cellular patterns in prostate cancer following various treatment modalities by performing texture and texture-derivative analysis on histology images. Specifically, we utilized GLCM [30], gray-level dependence matrix (GLDM) [31], gray-level size zone matrix (GLSZM) [32], and neighborhood gray-tone difference matrix (NGTDM) texture methods. Our findings demonstrate significant differences in cellular structure within the damaged regions after different types of treatment, highlighting the utility of radiomics texture analysis for quantifying histological changes and potentially guiding future therapeutic strategies.

## 2. Materials and Methods

### 2.1. Animal Model and Experimental Design

All animal procedures were conducted in accordance with the Canadian Council on Animal Care guidelines and were approved on 18 February 2025 by the Sunnybrook Research Institute Animal Care Committee (SRI ACC, protocol no. 539). The reporting of this study conforms to the ARRIVE 2.0 guidelines. Prostate cancer xenografts were established in New Zealand White male rabbits (Charles River Laboratories, Montreal, QC, Canada). Animals received daily intramuscular injections of cyclosporine (50 mg/mL; Sandimmune) (Novartis, Dorval, QC, Canada) as an immunosuppressant. This was performed three days prior to cell injection and continued until the day animals were scarified. Once the rabbits reached a body weight of 1.5–2.0 kg, anesthesia was induced with intramuscular ketamine (50 mg/kg) and xylazine (5 mg/kg). Subsequently, 8 × 10^6^ PC3 human prostate cancer cells (ATCC CRL1435) (Manassas, VA, USA), suspended in 500 µL phosphate-buffered saline (PBS), were injected into the right hind leg. PC3 cells were cultured in RPMI 1640 medium (Wisent Bio-Centre, St-Bruno, QC, Canada) supplemented with 10% fetal bovine serum (FBS) and 1% penicillin/streptomycin (Thermo Fisher Scientific, Waltham, MA, USA) and maintained at 37 °C in a 5% CO_2_ atmosphere prior to injection. Tumors were allowed to grow to a size of 1.5–2.0 cm, typically within 7–10 days after arrival at the research facility. These sizes were obtained over a period of 3–4 weeks.

Animals were randomized into six treatment groups, including (i) no treatment (control, *n* = 5), (ii) 8 Gy radiation (XRT, *n* = 4), (iii) ultrasound-stimulated microbubbles (USMBs, *n* = 5), (iv) combined ultrasound-stimulated microbubble and radiation (USMB + XRT, *n* = 5), (v) combined treatment for whole tumors and additional ultrasound-stimulated microbubble treatment to low-perfusion areas (USMB + XRT (LOW), *n* = 5), and (vi) combined treatment for whole tumors and additional ultrasound-stimulated microbubble treatment to high-perfusion areas (USMB + XRT (HIGH), *n* = 5).

Low- and high-perfusion regions were identified using dynamic contrast-enhanced magnetic resonance imaging (DCE-MRI). Pre-treatment DCE-MRI scans were analyzed to design pulse sequences targeting areas of differing vascularity for the USMB + XRT (LOW) and USMB + XRT (HIGH) groups. A maximum intensity projection (MIP) was generated from the normalized initial area under the DCE-MRI curve, and exclusion masks were created to target perfusion-specific regions. Specifically, 1.2 mm wide treatment cells containing voxels within the highest or lowest third of MIP values were excluded from the low- and high-perfusion targeted pulse sequences, respectively. Treatment-specific details follow.

### 2.2. Ultrasound Microbubble Treatment

For USMB, the Sonalleve bed and transducer were used with software designed for low-power microbubble stimulation. Definity microbubbles (Lantheus Medical Imaging, North Billerica, MA, USA) were activated for 45 s with a Vialmix device. A quantity of 2 mL of microbubbles was then diluted with 4 mL of saline and injected intravenously into the ear using a power injector, followed by a 3 mL saline + 0.2% heparin flush. Ultrasound was delivered via a transducer with an 800 kHz central frequency. Ultrasound was emitted at a peak negative pressure of 750 kPa using a pulse sequence with a 16-cycle tone burst and a 31 kHz pulse repetition frequency.

For animals receiving additional treatment to a particular area of the tumor (USMB + XRT (LOW), USMB + XRT (HIGH)), an additional 7 min pulse sequence was designed as shown in order to deliver additional USMB treatment to areas of low or high perfusion. This doubled the amount of USMB treatment. A decision was made to arbitrarily treat the 2/3 tumor volume that had the lowest perfusion (USMB + XRT (LOW)) or the highest perfusion (USMB + XRT (HIGH)). Dynamic contrast-enhanced MRI (DCE-MRI) was used to identify low- and high-perfusion regions within the single dose of combined USMB + XRT treated tumor region.

The pre-treatment anatomical MRIs (3 T MRI Philips Achieva, Philips Healthcare) were used to define an analysis region (18 mm diameter) in T1-weighted images, which was sub-divided into 1.2 mm cells. DCE-MRI of signal changes following contrast agent injection was analyzed semi-quantitatively to measure the percentage signal enhancement [33]. The curve was normalized to a signal in blood vessels or a reference tissue like muscle to correct the variation due to contrast agent administration or scan parameters. For each voxel in the treatment region, the area under the DCE-MRI curve (AUC) during the first two minutes after injection was calculated by subtracting the pre-contrast baseline signal intensity. To account for possible variations in the injection protocol, a normalized AUC (AUC_norm_) was also calculated by dividing the AUC in the tumor by that in a muscle region.

**Figure 1:**
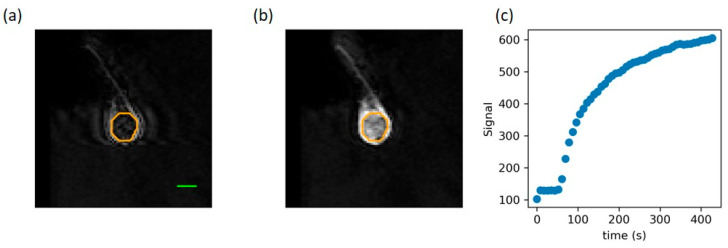
DCI-MRI images of a pre-treatment tumor (**a**) before injection and (**b**) 2.5 min after injection. (**c**) The mean signal intensity over the whole tumor increases following injection. The orange boundary indicates the tumor region of interest used to calculate the signal intensity. The green scale bar represents 1 cm.

A maximum intensity projection (MIP) of this initial AUC was then performed across the transverse slices. For pulse sequences targeting low-perfusion regions (USMB + XRT (LOW)), the third of voxels with the highest values in the MIP formed an exclusion mask (66% of tumor volume with the lowest perfusion treated). Any treatment cells with an excluded voxel were excluded from sonication. For pulse sequences targeting high-perfusion regions (USMB + XRT (HIGH)), the same procedure was performed but excluding the third of voxels with the lowest values in the MIP (66% of tumor volume with the highest perfusion treated). Figure 1 demonstrates the pulse sequence design procedure for animals that received targeted treatment to a low-perfusion area. Cells containing voxels that demonstrated high values on the MIP of the AUC were excluded from treatment for enhancement of the low-perfusion area (USMB + XRT (LOW)) and vice versa for treatments to enhance the high-perfusion areas (USMB + XRT (HIGH)).

### 2.3. Radiation Treatment

For XRT, rabbits were treated with a single dose of 8 Gy using a 160 kVp cabinet irradiator (CP160 X-Ray Irradiation System, FaxitronBioptics, Tucson, AZ, USA). To protect non-tumor tissues, the leg was covered with a 3 mm thick lead sheet, with a circular cut-out exposing the tumor. This radiation dose was selected given its known vascular effects. The dose rate was 100 cGy/min.

Twenty-four hours post-treatment, animals were euthanized and tumors were excised for histological analysis. Tumors were fixed in 10% formalin for 24–48 h, processed, and sectioned at 50 μm intervals. Sections were stained with hematoxylin and eosin (H&E) and Factor VIII. Histological images were acquired at 25× magnification using a TissueScope LE Scanner (Huron Digital Pathology, St. Jacobs, ON, Canada). Vascular density was calculated from Factor VIII histology images. Radiomics texture and texture-derivative parameters were extracted from whole-tumor, as well as from manually segmented tumor cells damaged regions from the H&E images. Staining of all tumor sections was performed at the same time to ensure consistency. Digital images were normalized.

### 2.4. Texture Analysis

Texture analysis was performed using four established methods, including (i) the gray-level co-occurrence matrix (GLCM), which quantifies the frequency of co-occurring pixel pairs with specific intensity values in defined spatial relationships within an image; (ii) the gray-level dependence matrix (GLDM), which measures the number of connected voxels within a certain distance that are dependent on the center voxel, capturing gray-level dependencies; (iii) the gray-level size zone matrix (GLSZM), which quantifies the size of homogeneous zones, defined as groups of connected voxels sharing the same gray level; and (iv) the neighboring gray-tone difference matrix (NGTDM), which calculates the difference between the intensity of a pixel and the average gray level of its eight surrounding pixels. 

For feature extraction, high-magnification H&E images were first down sampled 10 times tumor and cell-damaged regions were manually segmented using custom software using MATLAB version 2020b, converted to grayscale, and saved for analysis. A total of 59 radiomics features were extracted from each region, comprising 24 GLCM, 16 GLSZM, 14 GLDM, and 5 NGTDM parameters.

**Figure 2:**
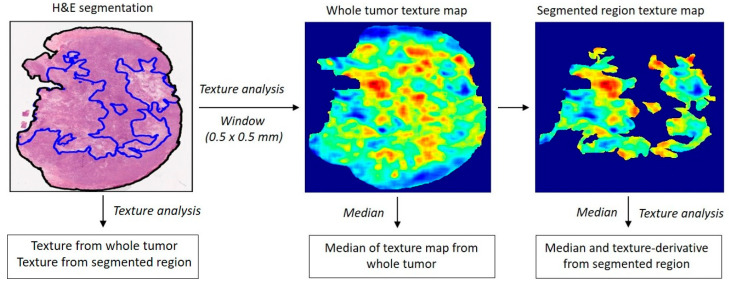
Flowchart: radiomics texture and texture-derivative parameter determination from the H&E histology images.

To assess local texture heterogeneity, a texture map was generated by dividing each segmented region of interest into 0.5 mm × 0.5 mm window blocks and performing texture analysis within each block. This approach visualizes spatial variations in texture patterns. Subsequently, a second-pass texture analysis was performed on the resulting texture maps to derive higher-order texture-derivative parameters. In total, 1053 texture-derivative features were extracted from each histology image. Diagrams of texture and texture-derivative parameter estimation from the histology images are shown in Figure 2.

### 2.5. Statistical Analysis

Statistical analyses were conducted using GraphPad Prism (La Jolla, CA, USA). Group comparisons were performed using one-way analysis of variance (ANOVA) followed by Bonferroni’s post hoc test for selected comparisons. A *p*-value of <0.05 was considered statistically significant, as indicated by lines above the compared groups in the figures. Each treatment group was compared with all other groups.

## 3. Results

Low-magnification hematoxylin and eosin (H&E)-stained images revealed pronounced degradation of the cellular architecture within tumor regions following treatment with USMB, XRT, combined USMB + XRT, USMB + XRT (LOW), and USMB + XRT (HIGH) treatments. The regions exhibiting cellular damage were manually delineated, and the fraction of the damaged area relative to the total tumor area was quantified. Notably, the combined USMB + XRT (LOW) and USMB + XRT (HIGH) groups demonstrated the most extensive cellular disruption. Compared to both the control and XRT-only groups, the fraction of tumor area exhibiting cellular damage was significantly higher in the combined USMB + XRT (LOW) and USMB + XRT (HIGH) groups, with a mean difference of 24.4 ± 4.2% (*p* < 0.0001, 95% CI: 10.7–38.1). Among all treatment arms, the combined USMB + XRT regimens produced the greatest extent of cell damage. Quantitative analysis of the H&E slides showed the following mean fractions of damaged areas: 11.2 ± 5.1% (control), 11.8 ± 0.6% (XRT), 29.4 ± 3.5% (USMB), 29.1 ± 2.2% (USMB + XRT), 36.9 ± 4.8% (USMB + XRT (LOW)), and 35.1 ± 3.9% (USMB + XRT (HIGH)).

**Figure 3:**
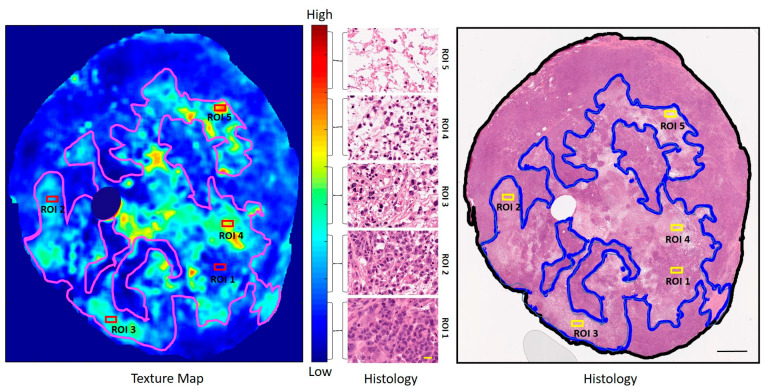
Whole GLDM-based *high-gray-level-emphasis* texture map corresponding to a representative PC3 treated with USMB + XRT (HIGH), with five regions of interest (ROI1, ROI2, ROI3, ROI4, and ROI5) from the lowest to highest parameter values, is displayed (**left**). Corresponding H&E histology and magnified images from those five ROIs are displayed (**right**). ROI1 and ROI2 show mostly tumor cells. ROI3 and ROI4 sections show a mixture of normal, condensed, and fragmented nucleus cellular structures. ROI5 has completely lost its cellular structure, exhibiting features consistent with patchy necrosis. The color bar represents the scale for the GLDM *high-gray-level-emphasis* parameter of 25 to 53. The ranges of the GLDM *high-gray-level-emphasis* parameter values for the ROI1, ROI2, ROI3, ROI4, and ROI5 regions are marked using the braces. The black bar in the low-magnification histology image is 2 mm, and the yellow bar in the high-magnification image is 30 µm.

Texture maps were generated to reflect distinct textural properties derived from the organization of cellular structures in H&E-stained sections (Figure 2). A representative *high-gray-level-emphasis (HGLE)* texture map, based on the GLCM method, is presented in Figure 3. Five regions of interest (ROI1, ROI2, ROI3, ROI4, and ROI5), deducting areas in the tumor with five different appearances that corresponded to low, intermediate, and high texture parameter values, were selected from the texture map and are presented compared with their respective histological images. Magnified views of these regions are shown in Figure 3. ROI1 and ROI2, characterized by the lowest *HGLE* values, predominantly displayed viable tumor cellular structures. ROI3 and ROI4, with intermediate *HGLE* values, exhibited a mixture of normal nuclei and condensed and fragmented dead nuclei representing early-stage apoptotic cell death structures. ROI5, representing the highest texture values, had completely lost its cellular structure and the cytoplasm had dissolved, indicating advanced cellular degradation.

### 3.1. Overall Texture Properties

**Figure 4:**
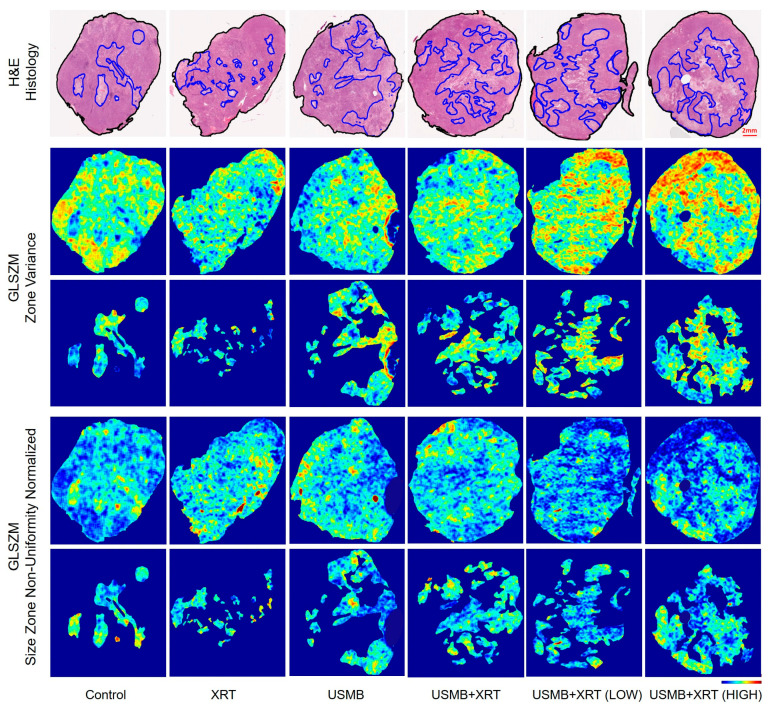
Representative H&E images from each treatment group showing manually segmented tumor affected regions and corresponding GLSZM0based *Zone Variance* (GLSZM_*ZV*) and GLSZM–*Size Zone Non-Uniformity Normalized* (GLSZM_*SZNN*) texture maps from whole-tumor and segmented regions. The color bar represents the scale for the GLSZM_*ZV* parameter of 0.07 to 0.12 and that for GLSZM_*SZNN* of 0.78 to 0.88. A lower GLSZM_*ZV* value indicates that the variation within the same gray-level zone sizes is small, while a higher value reflects greater variation in zone sizes. Similarly, a lower GLSZM_*SZNN* value represents greater uniformity in zone-size distribution, whereas a higher value indicates greater non-uniformity in the distribution of zone sizes. The red scale bar in the low-magnification histology image is 2 mm.

The methodology was applied to whole-tumor sections and slides. Representative H&E-stained histology images from both the whole-tumor and segmented cell death regions (outlined in blue) for each treatment group, including control, XRT, USMB, combined USMB + XRT, USMB + XRT (LOW), and USMB + XRT (HIGH), are presented in Figure 4. Qualitative assessment of these images revealed marked differences in cellular architecture among the various treatment groups. To quantitatively characterize these differences, a comprehensive texture analysis of the histology images was performed, extracting features from both the entire tumors and the segmented regions of cell death.

**Figure 5:**
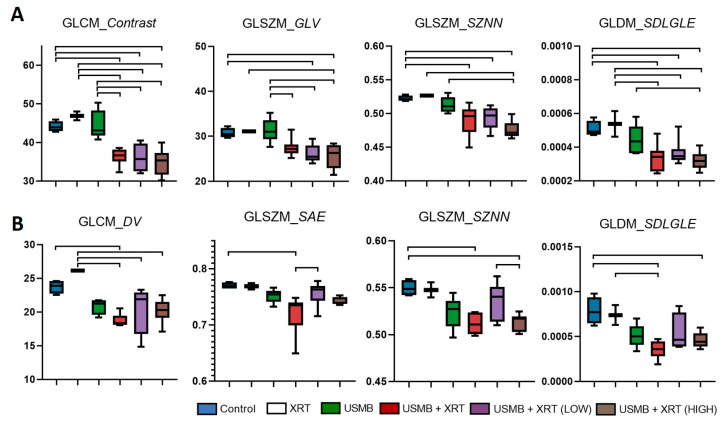
Radiomics texture parameters from H&E histology images for whole-tumor (**A**) and segmented regions (**B**). The top lines represent significant differences between two groups, *p* < 0.05. GLSZM_*GLV*: GLSZM *Gray-Level Variance*; GLSZM_*SZNN*: GLSZM *Size Zone Non-Uniformity Normalized*; GLDM_*SDLGLE*: GLDM *Small Dependence, Low Gray-Level Emphasis*; GLCM_*DV*: GLCM *Difference Variance*; GLSZM_*SAE*: GLSZM *Small Area Emphasis*.

Statistical analysis of the extracted texture parameters demonstrated significant differences between treatment groups, as illustrated in Figure 5. Specifically, Figure 5A displays results from the whole tumors, whereas Figure 5B presents results from the segmented cell death regions. Texture features derived from the GLCM, GLSZM, and GLDM demonstrated statistically significant variation across treatment groups. Notably, the GLCM contrast parameter, calculated from the whole-tumor region, was significantly lower in the combined treatment groups (USMB + XRT, USMB + XRT (LOW), and USMB + XRT (HIGH)) compared to the control, XRT, and USMB groups. Other GLCM-derived features, including *Difference Average*, *Difference Entropy*, and *Difference Variance*, as well as the GLSZM *Difference Average*, exhibited similar trends. These features, which are highly correlated (*R*^2^ > 0.8), are indicative of increased texture disorder within the tissue.

Further, GLSZM-based parameters, such as *Gray-Level Variance* (*p* < 0.0001, *R*^2^ = 0.568) and *Size Zone Non–Uniformity Normalized* (*p* < 0.0001, *R*^2^ = 0.568), were reduced in the combined treatment groups, reflecting decreased variation in gray-level intensities and greater uniformity in zone-size distributions. The GLDM feature *Small Dependence, Low Gray–Level Emphasis* (*p* < 0.0001, *R*^2^ = 0.568) was also significantly lower in the combined treatment groups, suggesting a reduction in the prevalence of small, low-intensity dependencies and thus a more homogeneous tissue texture. In contrast, GLCM-based *Inverse Difference Normalized* (*p* < 0.0001, *R*^2^ = 0.568) and *Inverse Difference Moment Normalized* (*p* < 0.0001, *R*^2^ = 0.568) were significantly higher in the combined treatment groups compared to the control, XRT, and USMB groups, indicating increased homogeneity in these images.

Analysis of the segmented cell death regions revealed similar patterns. Both GLSZM *Size Zone Non-Uniformity Normalized* (*p* < 0.0001, *R*^2^ = 0.568) and GLDM *Small Dependence, Low Gray-Level Emphasis* (*p* < 0.0001, *R*^2^ = 0.552) were lower in the USMB + XRT and USMB + XRT (HIGH) groups compared to the control and XRT groups. Additionally, GLCM *Difference Variance* (*p* = 0.0009, *R*^2^ = 0.495), another marker of texture disorder, was reduced in the combined treatment groups within the segmented regions. Interestingly, two GLSZM features—*Small Area Emphasis* (*p* = 0.0022, *R*^2^ = 0.427), which measures the distribution of small size zones, and *Size Zone Non-Uniformity Normalized* (*p* = 0.0022, *R*^2^ = 0.535)—were significantly higher in the USMB + XRT (LOW) group compared to the USMB + XRT and USMB + XRT (HIGH) groups.

Collectively, these findings demonstrated that combined USMB and XRT treatments induced significant and quantifiable alterations in tissue texture, as measured by multiple complementary texture analysis methods. These changes were consistent across both whole-tumor and segmented cell death regions, underscoring the potential of texture features as sensitive biomarkers for treatment response in histopathological analysis.

### 3.2. Local Texture Properties

**Figure 6:**
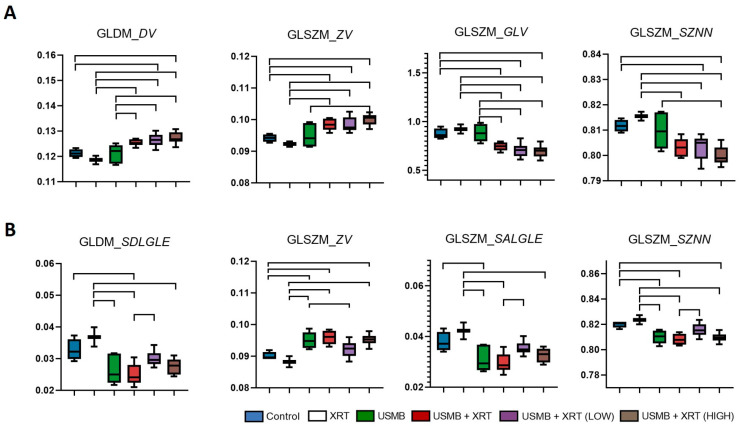
Median values of texture maps from H&E histology images for whole-tumor (**A**) and segmented regions (**B**). Top lines represent significant differences between two groups, *p* < 0.05. GLDM_*DV*: GLDM *Dependence Variance*; GLSZM_*ZV*: GLSZM *Zone Variance*; GLSZM_*GLV*: GLSZM *Gray-Level Variance*; GLSZM_*SZNN*: GLSZM *Size Zone Non-Uniformity Normalized*; GLDM_*SDLGLE*: GLDM *Small Dependence, Low Gray-Level Emphasis*; GLSZM_*SALGLE*: GLSZM *Small Area, Low Gray-Level Emphasis*.

In order to investigate local texture patterns within both the whole-tumor and the segmented cell death regions, texture maps were generated from the histology images using a windowing technique based on the GLCM, GLSZM, GLDM, and NGTDM methods. Representative H&E-stained histology, as well as GLSZM-based *Zone Variance* (*p* < 0.0001, *R*^2^ = 0.596) and *Size Zone Non-Uniformity Normalized* (*p* < 0.0001, *R*^2^ = 0.553) texture maps from both the whole-tumor and segmented regions for each treatment group, are shown in Figure 4. Visual assessment revealed clear differences in the texture patterns between the treatment groups, in both the whole-tumor and the segmented regions. The median values derived from these texture maps for all treatment groups are summarized in Figure 6.

As depicted in Figure 6A, the control, XRT, and USMB treatment groups exhibited greater variation in local gray values, with minimal changes in zone size. In contrast, all combined treatment groups showed reduced variation in gray values but increased variation in zone sizes. Notably, significant differences in these features were observed within the segmented regions among the treatment groups (Figure 6B). Unlike the whole-tumor analysis, local texture parameters in the segmented regions were significantly different in the USMB group compared to the control and XRT groups, while no significant differences were observed between the USMB + XRT and USMB + XRT (HIGH) groups. Furthermore, certain features, such as variation in zone size and uniformity of zone size, were significantly different in the USMB + XRT (LOW) group compared to the other combined treatment groups.

### 3.3. Intra-Tumoral Texture Properties

**Figure 7:**
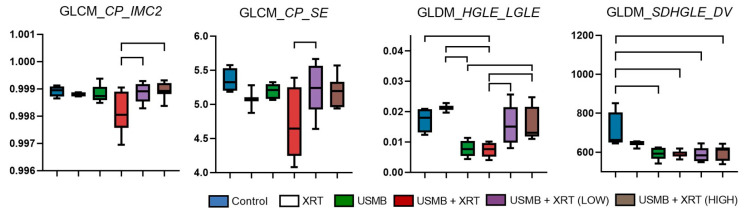
Texture-derivative values from H&E histology images for segmented regions. Top lines represent significant differences between the groups, *p* < 0.05. GLCM_*CP_IMC2*: GLCM *Cluster Prominence–Informational Measure of Correlation 2*; GLCM_*CP_SE*: GLCM *Cluster Prominence–Sum Entropy*; GLDM_*HGLE_LGLE*: GLDM *High Gray-Level Emphasis–Low Gray-Level Emphasis*; GLDM_*SDHGLE_DV*: GLDM *Small Dependence, High-Gray-Level-Emphasis-Dependence Variance*.

To further characterize intra-tumoral texture heterogeneity and any patters in textural features, derivative texture parameters were extracted from the texture maps using a second-pass texture analysis approach. Out of 1053 features analyzed, only a small subset exhibited significant differences between the treatment groups. The most notable significant features are presented in Figure 7. Specifically, the GLCM-derived features, *Cluster Prominence–Informational Measure of Correlation 2* (*p* = 0.0078, *R*^2^ = 0.366) and *Cluster Prominence–Sum Entropy* (*p* = 0.0199, *R*^2^ = 0.322), as well as the *GLDM-derived High Gray-Level Emphasis–Low Gray-Level Emphasis* (*p* = 0.002, *R*^2^ = 0.527), were significantly lower in the standard USMB + XRT treatment group compared to the USMB + XRT (LOW) and USMB + XRT (HIGH) groups.

## 4. Discussion

Ultrasound-stimulated microbubble (USMB) therapy, particularly when combined with radiotherapy (XRT), has emerged as a promising strategy for targeting tumor vasculature and enhancing the efficacy of conventional cancer treatments [14]. Recent preclinical studies, including those utilizing commercial MRI-guided focused ultrasound systems, have demonstrated that USMB can sensitize tumor vasculature to radiation, resulting in significantly greater tumor cell kill compared to radiation alone [16]. Dynamic contrast-enhanced MRI (DCE-MRI) has played a pivotal role in the present study by enabling the identification of low- and high-perfusion areas within tumors. This functional imaging technique provides detailed insights into tumor vascularity and perfusion, which are critical for guiding targeted therapies such as USMB and XRT. By mapping perfusion heterogeneity, DCE-MRI was used here for the precise delivery of additional USMB and XRT treatments to regions potentially most in need of vascular disruption, thereby maximizing therapeutic impact. Texture analysis is a powerful radiomics tool that extracts quantitative features from histology images, capturing subtle changes in tissue architecture that may not be apparent through conventional histopathological assessment. In the current study, four advanced texture analysis methods, GLCM, GLDM, GLSZM, and NGTDM, were used to quantify changes in the cellular structure of prostate tumors following various treatment regimens, including 8 Gy XRT, USMB, USMB + XRT, USMB + XRT (LOW), and USMB + XRT (HIGH).

The results revealed that most texture features, particularly those derived from GLCM, GLDM, and GLSZM, exhibited significant differences in cell structure patterns across treatment groups. In the whole-tumor regions, GLCM and GLSZM intensity-based texture disorder parameters were significantly lower in all combined treatment groups compared to the control, XRT, and USMB-only groups (Figure 5). This suggests a reduction in textural disorder and a shift toward a more homogeneous cellular architecture following combined therapy, likely due to the development of cell death throughout tumor sections. Additionally, the variation in gray zone size was higher and more uniformly distributed in the combined treatment groups, reflecting the presence of clusters at different stages of cell death, including late-stage cell death, necrosis, and loss of glandular structure. A similar trend in texture parameters was observed within the segmented regions. However, only the USMB + XRT and USMB + XRT (HIGH) treatment groups showed a significant difference in zone-size distribution parameters compared to the control and XRT groups. This suggests that, within the segmented regions, the USMB + XRT (LOW) treatment group may contain a higher proportion of early-stage cell death areas compared to late-stage regions, or vice versa.

Local texture patterns, assessed by calculating the median value from texture maps, mirrored the trends observed in the overall analysis. GLDM-based *Dependence Variance* and GLSZM-based *Zone Variance* were significantly different in the combined treatment groups compared to the control and XRT groups, indicating greater variation in pixel dependence size and zone size. Statistical analysis of the median values revealed that combined treatment groups exhibited more homogeneous structural patterns at the gray-value level, but greater heterogeneity at the zone-size level, compared to the control, XRT, and USMB groups (Figure 6). This suggests that the cellular pattern differences induced by combined therapy are captured not only globally but also at the local level. Interestingly, within segmented regions, these local texture differences were not as pronounced, except for the USMB group, which showed significant differences compared to the control and XRT groups. This finding implies that USMB treatment alone can induce substantial changes in tumor cellular structure, which may not be fully captured by overall texture parameter estimation. Moreover, the local texture patterns in the USMB group closely resembled those in the combined treatment groups, both at the pixel and zone levels.

Texture-derivative parameters, estimated from texture maps, provided further insights into intra-tumoral heterogeneity. These can be related to spatially repeating textural features or changes in higher-order cellular organization patterns. Pixel-related GLCM and GLDM texture-derivative parameters, particularly those derived from *cluster prominence* and *high-gray-level-emphasis* maps, showed significant differences between additional USMB + XRT treatment groups and single-dose USMB + XRT groups (Figure 7). The intra-tumoral cellular structural properties reflected by these parameters were significantly higher in regions treated with additional USMB + XRT compared to single-dose combined treatment and were similar to those observed in the control and XRT groups. This highlights the sensitivity of texture-derivative analysis in detecting subtle changes in intra-tumoral architecture induced by different treatment intensities.

The findings of this study are consistent with previous research in other cancer types, such as breast cancer, where texture analysis of QUS parametric maps has been successfully used to monitor treatment response and predict outcomes. In these studies, the integration of texture features with QUS parameters significantly improved the performance of treatment response prediction models [28]. Similarly, in the current prostate cancer model, texture analysis—particularly using GLSZM—proved highly effective in detecting structural changes associated with combined USMB and XRT treatments. Among the four texture analysis methods evaluated, GLSZM stood out as the most sensitive and robust for capturing treatment-induced changes in tumor structure. GLSZM quantifies the size and distribution of homogeneous gray-level zones, making it particularly well-suited for identifying regions of necrosis, fibrosis, or varying stages of cell death within tumors. This capability is crucial for assessing the efficacy of therapies that target tumor heterogeneity and induce complex patterns of cell death. However, due to the small sample size in each treatment group, the texture parameters extracted from both the whole-tumor and the cell-damaged regions did not show significant differences between the individual treatment groups. Increasing the sample size in future studies should improve the ability to discriminate between groups. Additionally, the cell-damaged regions were segmented exclusively by our highly experienced research technician. In the future, incorporating multiple observers will help to emphasize and assess the reproducibility of the segmentation processes. Recently, deep learning techniques have emerged as promising tools for the automated segmentation and differentiation of various tissue types in histology images [34,35]. These methods have demonstrated a performance comparable to that of expert pathologists in tasks such as tumor detection and tissue-type identification, significantly reducing the time and effort required for image analysis. Building on these advances, we are currently expanding our study to investigate the effectiveness of several pre-trained deep learning models in identifying changes in cellular structure in histology images. This approach aims to further enhance the accuracy and efficiency of tissue characterization in digital pathology.

## 5. Conclusions

In summary, this study demonstrates that changes in cellular structure patterns induced by focused ultrasound and radiation exposure can be effectively quantified using advanced texture analysis methods—GLCM, GLDM, GLSZM, and NGTDM—applied to histology images. Among these, the GLSZM methodology emerged as the optimal method for representing structural changes in tumors following treatment. Texture-derivative techniques further enabled the detection of intra-tumoral heterogeneity, particularly in response to additional USMB and XRT treatments targeted to low- and high-perfusion regions. These findings highlight the potential of texture analysis as a powerful tool for monitoring cancer treatment response, particularly when combined with ultrasound-stimulated microbubbles and radiotherapy, which target tumors through an anti-vascular mechanism.

## Data Availability

The raw data for this project can be made available by Dr. Gregory J. Czarnota upon request.

## Abbreviations

The following abbreviations are used in this manuscript:

USMB	Ultrasound-stimulated microbubble
XRT	Radiotherapy
DCE-MRI	Dynamic contrast-enhanced magnetic resonance imaging
GLCM	Gray-level co-occurrence matrix
GLDM	Gray-level dependence matrix
GLSZM	Gray-level size zone matrix
GLRLCM	Gray-level run-length co-occurrence matrix
NGTDM	Neighborhood gray-tone difference matrix
ROI	Region of interest
H&E	Hematoxylin and eosin
